# Hydrogen Ion Dynamics as the Fundamental Link between Neurodegenerative Diseases and Cancer: Its Application to the Therapeutics of Neurodegenerative Diseases with Special Emphasis on Multiple Sclerosis

**DOI:** 10.3390/ijms23052454

**Published:** 2022-02-23

**Authors:** Salvador Harguindey, Khalid Alfarouk, Julián Polo Orozco, Stephan J Reshkin, Jesús Devesa

**Affiliations:** 1 Division of Oncology, Institute of Clinical Biology and Metabolism, 01004 Vitoria, Spain; polorozco@gmail.com; 2 Institute of Endemic Diseases, University of Khartoum, Khartoum 11111, Sudan; alfarouk@hala-alfarouk.org; 3 Department of Biosciences, Biotechnology and Biopharmaceutics, University of Bari, 70125 Bari, Italy; stephanjoel.reshkin@uniba.it; 4 Scientific Direction, Foltra Medical Centre, 15886 Teo, Spain; jesus.devesa@usc.es

**Keywords:** neurodegenerative diseases—multiple sclerosis, new therapeutic options for multiple sclerosis and other neurodegenerative diseases, pH in cancer and neurodegenerative diseases—cancer and neurodegeneration as opposed processes—metabolic etiopathogenesis of cancer and human neurodegenerative diseases

## Abstract

The pH-related metabolic paradigm has rapidly grown in cancer research and treatment. In this contribution, this recent oncological perspective has been laterally assessed for the first time in order to integrate neurodegeneration within the energetics of the cancer acid–base conceptual frame. At all levels of study (molecular, biochemical, metabolic, and clinical), the intimate nature of both processes appears to consist of opposite mechanisms occurring at the far ends of a physiopathological intracellular pH/extracellular pH (pHi/pHe) spectrum. This wide-ranging original approach now permits an increase in our understanding of these opposite processes, cancer and neurodegeneration, and, as a consequence, allows us to propose new avenues of treatment based upon the intracellular and microenvironmental hydrogen ion dynamics regulating and deregulating the biochemistry and metabolism of both cancer and neural cells. Under the same perspective, the etiopathogenesis and special characteristics of multiple sclerosis (MS) is an excellent model for the study of neurodegenerative diseases and, utilizing this pioneering approach, we find that MS appears to be a metabolic disease even before an autoimmune one. Furthermore, within this paradigm, several important aspects of MS, from mitochondrial failure to microbiota functional abnormalities, are analyzed in depth. Finally, and for the first time, a new and integrated model of treatment for MS can now be advanced.

## 1. Introduction

As a classical approach to disease in general [1], the concept of homeostasis was initially defined as a balanced and healthy physiological state of the cells, tissues, and systems of the organism. During the first part of the 20th century, Walter Cannon and Hans Selye initiated the seminal studies on the concept of homeostasis. However, the concept of acid–base balance was initially disregarded from belonging to the homeostatic approach [2]. These precedents inspired Hans Selye to create his famous General Adaptation Syndrome, a dynamic mixture of homeostasis and allostasis [3]. Later on, these concepts spread out to include acid–base equilibrium and disequilibrium within its pathophysiological range. A healthy state of local and systemic homeostasis is fundamental for the correct functioning of all cells, organs, and tissues of the organism. This requires that its normal parameters are kept within very narrow limits. The new pH-centric anticancer paradigm [4,5] can now be applied as an extension of the field of oncology into the field of neurodegeneration. This translational and transversal research among vastly separate fields will allow for a better understanding of the intimate nature of the pH-related deregulation of this homeostatic pH-related approach, showing that cancer and neurodegeneration are two opposed situations on an acid–base and energetic homeostatic spectrum. This becomes evident no matter at which level of analysis (either molecular, metabolic, biochemical, or even clinical [6,7,8,9]) this is approached (Table 1). Indeed, this perspective permits a deeper understanding of the intimate nature of both disease processes.

Regarding etiopathogenesis, the intracellular–extracellular hydrogen ion (H^+^) and/or pH dynamics, and its abnormalities, as the fundamental link between cancer and human neurodegenerative diseases (HNDDs) is presented in Table 2.

**Table 2:** pH-related mechanisms in the etiopathogenesis of multiple sclerosis (MS), human neurodegenerative diseases (HNDDs), and cancer. Abbreviations: AD, Alzheimer’s disease; PD, Parkinson’s disease; BC, breast cancer; IOs, ion channels; ASIC1, acid-sensing ion channel type 1a; Hv1, voltage-gated proton channel type 1.5; Nav1.5, voltage-gated sodium channel isoform 1.5; TME, tumor microenvironment; EC, extracellular space; GFs, growth factors; PDGF, platelet-derived growth factors.

Mechanism	Etiopathogenesis	References
Microenvironmental acid pH	As in all malignant tumors, a microenvironmental acidic pH is a fundamental hallmark of the demyelinating lesions of MS and other HNDDs. This pathologically acidified pHe decreases the migration, proliferation, and survival of oligodendrocyte precursor cells; hinders the cell differentiation into mature oligodendrocytes; and induces demyelination while decreasing remyelination. In AD, accumulation of β-amyloid (βA) is directly induced by acidosis. In PD, low neural cell pH induces protein aggregation, mitochondrial dysfunction, oxidative stress, and neuroinflammation, all hallmarks of the disease. Low pHi also activates pHi-dependent caspases and endonucleases.	[8,10,11,12,13,14,15,16,17,18,19,20,21,22,23,24,25,26,27,28,29,30,31]
H^+^ extrusion and elevated pHi	H^+^ extrusion on its own is a fundamental carcinogenic factor that induces cell transformation, growth, and invasion in BC and other tumors. On the contrary, H^+^ extrusion is an anti-apoptotic process in MS and HNDDs.	[6,7,8,32,33,34,35,36,37,38,39,40,41,42,43,44]
Ion channels (IOs) in HNNDs pathogenesis	IOs, mainly the isoform ASIC1, favor an i.c. excessive accumulation of Na^+^, Ca^++^, and H^+^ in MS and other HNDDs, resulting in severe axonal degeneration and neural damage, secondary to Ca^++^ overload and acidification-mediated apoptosis. Tissue acidosis further activates ASIC1, which precedes neuroinflammation and other autoimmune phenomena. A decrease in the CNS pHi opens ASIC1, which, through the stimulation of Ca^++^ into neural cells, induces axonal injury, apoptosis, and demyelination in MS, and β-amyloid accumulation in AD.	[45,46]
Ion channels (IOs) in cancer pathogenesis	Different ion channels (IOs) are involved in the deregulation of the pHi/pHe system in cancer cells, stimulating cell proliferation, matrix invasion, resistance to apoptosis, and metastatic potential. Hv1 and/or Nav1.5 have been found to be highly expressed in highly invasive BC cells, but not in poorly invasive BC cells.	[10,11,47,48,49,50,51,52,53]
Acidosis and immunity	Acidity of the tumor microenvironment (TME) disrupts the body immune defense mechanisms towards malignant tumors, locally and systemically. This allows relentless and uncontrolled tumor progression. Neutralizing tumor EC acidity with alkaline solutions improves the immune response.	[54,55,56,57,58]
Human growth factor (GFs) abnormalities in HNDDS and cancer	Removal of essential GFs result in apoptosis. NHE activity is fundamental in the pH regulation of the CNS, normalizing neural homeostasis by stimulating cellular metabolism and DNA synthesis. PDGF has been shown to induce an important decrease in brain amyloid-β (Aβ) deposition and tau phosphorylation in a mice model of AD, also reducing inflammatory responses and promoting Aβ degradation.	[6,7,26,59,60,61,62,63,64]
Mitochondriopathy in MS and HNDDs.	Mitochondrial dysfunction has been considered to represent a significant etiopa-thogenic factor in the pathogenesis and progression of several HNDDs. Mitochondria also play a crucial role in oligodendrocyte differentiation. Any perturbation in mitochondrial function is likely to damage myelinogenesis and worsen the evolution of MS.	[65,66,67,68,69,70]
Lactic acid (LA) in MS and HNDDs	LA levels are higher in MS patients as compared to healthy individuals. LA levels also become elevated with disease activity, progression, and/or during relapses. Cerebrospinal fluid (CSF) LA concentrations show a close link between MS plaque activity and LA metabolism.	[71,72]
RNMDA receptors in HNDDs	Glutamate is the primary excitatory neurotransmitter of the CNS and has a central role in the communication network between neurons, astrocytes, oligodendrocytes, and microglia. While glutamate induces multiple beneficial and essential effects, excess glutamate is catastrophic.	[73,74,75,76]
Microbiota in the etiopathogenesis of MS and HNDDs	Gut dysbiosis increases intestinal permeability and impairs Treg cell function, leading to inflammation and oxidative stress.	[77,78]

### 1.1. On pH-Related Etiology and Pathogenesis of Cancer in the Post-Warburg Era

Basic and clinical research into the regulation and deregulation dynamics of the hydrogen ion (H^+^), the intracellular pH (i.c. or pHi), and extracellular pH (e.c. or pHe) in health and disease has grown at an increasingly fast rate since the 1970s in many areas of medicine and science. While the dynamics of the hydrogen ion (H^+^) initially interested the realm of physiology, other areas of medicine, mainly oncology and neurology, were progressively included within this new and integral perspective [4,6,9,10].

Lately, the study of the dynamics of the H^+^ in medical research, mainly in cancer, has been extended sideways to include areas from the field of theoretical physics [79] to, most recently, the pathogenesis of schizophrenia [80]. This contribution also aims to revive and actualize these classical seminal concepts on systemic homeostasis and physiopathology, in order to apply them to the fields of neurology and oncology at a cellular and microenvironmental level. These concepts will mainly focus upon the etiopathogenesis of human neurodegenerative diseases (HNDDs), mainly multiple sclerosis (MS), and their links and differences with cancer, all in order to implement new therapeutic possibilities in both areas based upon an integral and homeostatic perspective. 

Regarding the cancer situation, complete listings of metabolically-oriented anticancer medications have been published by our group [4]. In that previous seminal publication on cancer and neurodegeneration, the emphasis rested on cancer treatment. In the present contribution, the main emphasis will rest on a metabolic approach to the etiopathogenesis and treatment of neurodegenerative diseases, with a special emphasis on MS.

**Figure 1:**
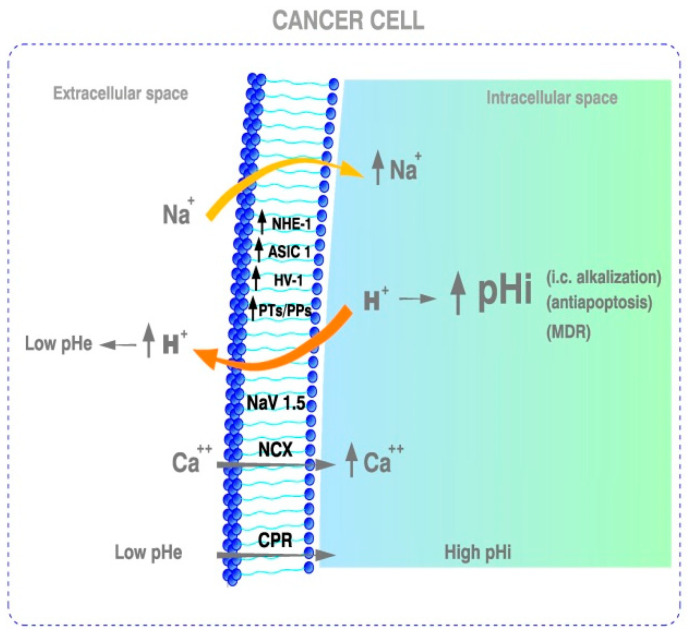
**Metabolic characteristics of cancer cells**. Intracellular alkalization of multiple upstream processes is the universal mediator of malignant transformation and the main metabolic and acid–base anti-apoptotic factor, which is also fundamental in MDR and chemotherapy. The secondary interstitial acidification of tumors (CPR) drives the following cascade in the metastatic process. Abbreviations: NHE1, Na^+^/H^+^ antiporter isoform 1; ASIC1, acid-sensing ion channel type 1a; Hv1, voltage-gated Na^+^ and H^+^ channel isoform 1; PTs, proton transporters; PPs, proton pumps; Nav 1.5, voltage-gated sodium channel isoform 1.5; NCX, Na^+^/Ca^2+^ exchanger; CPR, cancer proton reversal; MDR, multiple drug resistance. For further details, see text.

Nowadays, the prime cause of cancer is no longer considered to be the aerobic glycolysis of tumors, as Otto Warburg defended all his life. Instead, the latest evidence indicates that **the ‘prime’ cause of cancer, thus the fundamental carcinogenic factor behind the induction of the aerobic glycolysis initially described by Warburg** [4,32], **is the selective pathological alkalization of cells in all malignant tumors and leukemias, which is induced by a wide array of mediating factors** [32,40]. The upregulation of the Na^+^/H^+^ exchanger isoform 1 (NHE1) has been demonstrated to be the main etiologic factor in oncogene-driven neoplastic transformation. In this case, the activation of NHE1 stimulates proton (H^+^) extrusion, with resultant microenvironmental intracellular alkalization and extracellular acidification [33,34,35]. Intracellular alkalization also appears as the primary driver of many other cancer hallmarks, including aerobic glycolysis, DNA synthesis, and tumor growth [33,36]. This high pHi/low pHe situation creates a cancer-selective and pathognomonic reversed H^+^ gradient, also known as cancer proton reversal (CPR), which is mediated by the highly pathological deregulation of H^+^ dynamics in all malignant cells and tissues (↑pHi/↓pHe) [34,37,38,81] (Figure 1).

Moreover, it has been shown that proton (H^+^) efflux, on its own, induces dysplasia and potentiates cancer cell growth and invasion by oncogenic Ras. On the contrary, inhibiting H^+^ efflux induces cell death in invasive tumor cells lines [32,39]. Highly important are the outstanding results obtained by the group of Fliegel, which have shown that NHE-mediated H^+^ extrusion alone has a direct carcinogenic effect on breast cells [41,42]. In these studies, NHE1 hyperactivity appears to be an early and decisive driver in breast cancer (BC) carcinogenesis [36]. Furthermore, an H^+^ efflux-driven increase in pHi induces the in situ progression from precancerous lesions to invasive BC [43]. In summary, this evidence further indicates that H^+^ efflux is the main transforming factor in metabolic carcinogenesis [34,37,38,81] (Table 1 and Figure 1)**.** Finally, while NHE1 upregulation and its extracellular consequences (low pHe and CPR) indicate that H^+^ is the fundamental mechanism behind fast pHi-driven tumor growth and metastatic progression, similar pH changes can also be mediated by other membrane-bound proton transporters (PTs), such as monocarboxylate transporters (MCTs), acid-sensing ion channel type 1 (ASIC1), and proton pumps (PPs) [44,82,83,84,85,86,87,88,89] (Figure 1).

### 1.2. Molecular, Biochemical, Metabolic, and Genetic Links and Differences between Neurodegeneration and Cancer

While one of the main hallmarks of malignant tumors is now considered to be the selective PCR described above [4], HNDDs systematically show a strong tendency towards proapoptotic acidification of the pHi. This is the opposite of the cancer situation, and is the main and all-pervasive metabolic difference between HNDDs and all cancers, from solid tumors to leukemias [4].

**Figure 2:**
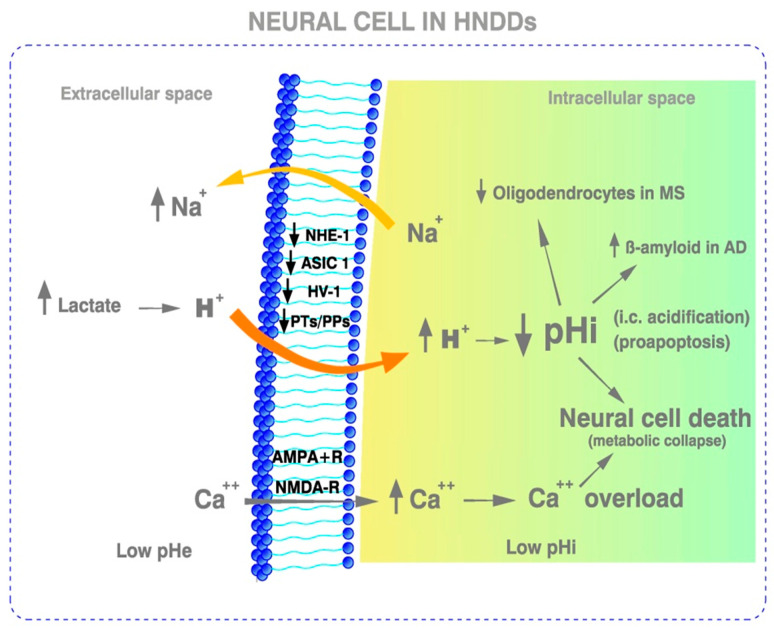
**Metabolic characteristics of HNDDs**. The downregulation of NHE1, ASIC1, Hv1, and/or proton transporters, together with a toxic intracellular Ca^++^ overload, produce further deviations towards acidification of neural cells. This is followed by activation of proteolytic cascades in neurons. A Ca^++^ overload such as this can be also mediated by an excessive release of glutamate, leading to the overactivation of the glutamatergic excitatory NMDA receptors. An opposite situation takes place in HNDDs than that in cancer cells regarding the pHi of cells. The low pHe in HNDDs can be secondary to an intracellular acidosis of a metabolic origin (metabolic/aerobic acidification) and/or to acidosis related to a lack of oxygen (hypoxic/ischemic/anaerobic acidosis). Abbreviations: HNDDs, human neurodegenerative diseases; AD, Alzheimer’s disease: MS, multiple sclerosis; NHE1, Na^+^/H^+^ exchanger isoform 1; ASIC1, acid-sensing ion channel type 1a; Hv1, voltage-gated proton channel type 1; Nav1.5, voltage-gated sodium channel isoform 1.5; PTs, proton transporters; PPs, proton pumps; NCX, Ca^++^/Na^+^ exchanger; CPR, cancer proton reversal; AMPA-R, α-amino-3-hyroxil-5-methyi-4-isoxazolepropionic acid receptor; NMDAR, N-methyl-d-aspartate receptor. For further details, see text.

Therefore, what can be therapeutic for cancer, i.e., the induction of a selective and proapoptotic pHi acidification, should be damaging to HNDDs (Table 1 and Figure 1 and Figure 2)**.** Furthermore, this dynamic pHi-centric perspective leads to a new and integral paradigm, and to a unified pH-related theory of the apoptosis–anti-apoptosis machinery mediated by the intracellular/extracellular hydrogen ion dynamics and related secondary pro-death and anti-death mechanisms. Interestingly, epidemiological studies have shown that Alzheimer’s disease and cancer exhibit an inverse association, once again indicating the opposed tendency of these two situations [90]. 

Other different perspectives linking cancer and neurodegeneration, such as the genetic mutation theory, have concluded that, while both processes share some genes and molecular mechanisms, they do so in opposite ways [91,92,93]. The genetic approach proposes that somatic mutations and DNA damage cause the disruption of the genetic homeostasis of cell populations in both cancer and HNDDs [94]. Furthermore, different genetic factors, such as the inheritance of mutated genes, have been proposed to facilitate the onset of cancer and neurodegeneration [92,95]. 

However, the genetic, immune, and metabolic approaches do not exclude each other. On the contrary, it seems possible that they can be integrated and, to a certain point, unified, following Nijhout’s metaphor: “*When a compound codified by a gene is needed, it is a signal from the environment which activates the expression of the gene and never an intrinsic characteristic of the gene*”. Indeed, the fact that genetic deregulations require mediating metabolic pathways to induce certain damaging effects has been repeatedly shown in the cancer context in different situations; for instance, Na^+^/H^+^ upregulation mediating different carcinogenic oncogenes and viruses [33,96,97]. These have also been found in certain cancer situations that have unified genetic factors with pH abnormalities and NHE deregulation in leukemias. Similarly, the integration of pH/NHE-related etiopathogenesis of certain lymphomas with immune parameters has also been reported (48). The remaining aim is to find similar specific cause–effect relationships among genetic deregulations, metabolic consequences, and immune disturbances in HNDD processes.

### 1.3. Multiple Sclerosis (MS) as an Excellent Model for the Study of HNDDs. The Million-Dollar Question on the Etiopathogenesis of MS Is: What Comes First, Immune or Metabolic Malfunctioning? Further Parallelisms and Differences with Cancer

MS is a disabling disease of unknown etiology in which the protecting layers of the peripheral nerves, the spinal cord, and the brain are affected. The capricious and sometimes unpredictable evolution of MS induces a myriad of multi-organ symptoms from its two main forms, the relapsing form or the steadily progressive form. In all cases, its evolution becomes progressively worse as the disease advances, creating increasing disability, and in many cases shortening the patient’s life.

During the last few years, many methods have been tried to improve our understanding of the essential nature of MS, as well as its prognosis and therapy. Nowadays, it is generally accepted that the fundamental nature of MS is characterized as a disorder of the immune system, with a secondary destruction of nerve sheaths through an apoptosis-dependent demyelination process [98]. 

As compared to other HNDDs, there is no doubt that immune abnormalities are most significant in the pathogenesis of MS. To date, there is no cure for MS; therefore, alternative approaches to treating this disease, and HNDDs in general, are necessary. Recently, B cell-depleting monoclonal antibodies, such as the anti-CD20 agent ocrelizumab, have shown a significant effect against MS, both in the relapsing form of the disease and, perhaps to a lesser degree, in slowing the relentless advance of the primary progressive form [99,100]. In addition, it is widely accepted that T cells are also involved in the pathogenesis of MS. Therefore, not only are CD-20-depleting therapies, such as ocrelizumab, available, there exist other differently acting agents that mainly affect T cells. However, during the last few years, some doubts have been raised concerning the immunological origin of MS [101]. 

The potential remyelinating effects of the substance cytidine-5′-diphospho (CDP)-choline was studied in murine models of MS. The effects of exogenously applied CDP-choline were tested, and increased remyelination was shown alongside an increase in the number of proliferating oligodendrocyte precursor cells and oligodendrocytes. Thus, CDP-choline could be a promising substance for patients with MS if used as a complementary therapy alongside other methods [102].

An acidic pHe, as in all malignant tumors, is also a fundamental characteristic of the demyelinating lesions in MS and other HNNDs (Table 1 and Figure 2). While an acidic pHe decreases the migration, proliferation, and survival of oligodendrocyte precursor cells, the fundamental effect seems to be that it also reduces their differentiation into mature oligodendrocytes, thus contributing to demyelination while hindering remyelination [15,103].

It is becoming increasingly recognized that metabolism and immunology are inextricably linked. Cellular metabolism causes immune cells to adopt different functions that depend of the state of the microenvironment, thus determining cellular life or death [104]. In cancer, the tumoral microenvironment is fundamental in regulating antitumor immunity; the characteristic pHe acidification allows tumors to escape the antitumoral immune response of the organism. In this situation, the final result of the intratumoral–interstitial (EC) low pHe is the creation of a protective shield, both around and within, malignant tumors, leading to a state of immunosuppression mediated by a low pHe-induced loss of function of T and NK cells [54,55]. On the contrary, neutralizing the tumor EC acidity with alkaline solutions improves antitumor responses to immunotherapy [56,57,58]. 

Along the same oncological line, Marches et al., showed the cause–effect relationship between metabolism and immunity by demonstrating that the anti-IgM-mediated induction of cell death in human B lymphoma cells (the immune response) is secondary, and dependent on the inhibition of the sodium/hydrogen exchanger (NHE1) and early pHi acidification (the metabolic response). Most importantly, these data allow the unification of two apparently vastly separate fields of research, metabolism and immunity, under one wide-ranging unit, referred to as “immunometabolism” [105]. This perspective indicates that the immune anti-IgM treatment depends on, and is secondary to, a metabolic NHE1-controlled pHi, and that the inactivation of the NHE1 in anti-IgM-stimulated cells results in cellular acidification that triggers apoptotic cell death. The conclusion is that, at least in these situations, metabolic changes precede the immunological [16,106]. Perhaps the term “immunometabolism” should be replaced, at least in such situations, by “metabolic immunity”, depending on which one of the two mechanisms is first at work [107,108]. 

Apart from the above considerations on the cause–effect interrelationships between metabolic and immunological disfunctions, we initially showed the similarities and differences between cancer and neurodegeneration at multiple levels, from basic research to clinical therapeutics [4,6,7]. Finally, in all the above-considered cases, metabolic abnormalities appear to precede immunological malfunctioning and cellular damage (“metabolic immunity-” or “immunometabolism-” dependent).

### 1.4. Cellular and Microenvironmental Acid–Base Abnormalities in Neurodegenerative Diseases

In order to maintain neuronal excitability, synaptic transmission, and neurotransmission in the CNS, the organism needs to keep their i.c. and e.c. microenvironmental acid–base homeostasis within very narrow limits [109,110]. A pathological lowering of pH takes place in MS and other HNDDs, where Ca^++^ overload can further promote i.c. acidification to induce neuronal proteolysis and apoptosis [111,112] (Figure 2). An acidified intra and extracellular pH is also pathognomonic of Alzheimer’s disease (AD), amyotrophic lateral sclerosis (ALS), and Huntington’s disease (HD), and is fundamental in the progression of these and other HNDDs [8,10,11,12,13,14]. A low pHi and pHe rapidly decreases neuronal transmission and activity, preceding the onset of neural cell death by apoptosis [17,18,19,20,21], the same response as induced by ASIC1 [22]. In the same vein, the accumulation of β-amyloid (βA) is directly induced by acidosis [11,12,13,14,16,17,18,19,20,21,22,110,112,113] (Figure 2 and Table 2). Furthermore, low neural cell pH induces protein aggregation, mitochondrial dysfunction, oxidative stress, and neuroinflammation, which are hallmarks of Parkinson’s disease [23]. The low pHe in PD and other HNDDs can be secondary, either to i.c. acidosis of a metabolic origin (metabolic/aerobic acidification) and/or to acidosis related to a lack of oxygen (hypoxic/ischemic/anaerobic acidosis) [23]. Finally, at least in ALS, i.c. acidification-mediated apoptosis is secondary to a Ca^++^-dependent effect, a pathogenetic mechanism considered to be responsible for disease progression [21,114] (Table 2). 

### 1.5. Acid–Base Dynamics and Homeostasis as the Main Factors Allowing a Better Understanding of the Intimate Nature of HNDDs and Cancer. Multiple Sclerosis as a Fundamnetal Basic Linking Model

Intracellular acidification is more pronounced in the brain of AD patients [11,113]. In a mice model of AD, βA aggregation was induced by acidosis and reverted upon microenvironmental alkalization, and the same occurs when using plasma rich in growth factors (PDGF) [16,21,24,25,26]. Lowering the pHi of neurons from 7.36 to 7.09/7.00 through exposure to nitric oxide (NO) sets in motion a programmed cell death program, increasing DNA fragmentation and decreasing cell survival (“low pHi-mediated metabolic collapse”) [7,8,11,27,28,29]. This low pHi and low pHe-mediated apoptosis results from the activation of three low pHi-dependent caspases and endonucleases [30,31]. Thus, maintaining a strict and narrow range of pHi/pHe homeostasis in the CNS becomes mandatory in neural protection, since acid–base parameters control a myriad of neuronal functions, including excitability, synaptic transmission, neurotransmitter uptake, intercellular communication, nociception, and inflammation [7,8,11,27,28]. Altogether, this data indicates that NO and caspase inhibition are theoretically indicated in AD and MS, and also in other HNDDs [30,31] (Section 2.11, Section 2.15 and Table 3).

**Table 3:** **Metabolically-based therapeutic options based upon the similarities and differences between cancer and neurodegeneration**. Abbreviations: Nav1.5, voltage-gated sodium channel isoform 1.5; ASIC, acid-sensing ion channel; NHE1, Na^+^/H^+^ exchanger isoform 1; MS: Multiple sclerosis; NHE1, Na^+^/H^+^ exchanger isoform 1; AM, amiloride; CP, cariporide; 4-AP, aminopyridine; NM, nafamostat mesylate; GFs, growth factors; PDGF, platelet-derived growth factors; PD, Parkinson’s disease; hGH: human growth hormone; AD, Alzheimer’s disease; ALS, amyotrophic lateral sclerosis; CSF, cerebrospinal fluid.

Mechanism	Therapeutic Options	References
Ion channels/ASIC inhibitors	NaV1.5 Na^+^ channels associate with NHE1 to become overexpressed in breast cancer, stimulating the formation of invadopodia and the metastatic process. The utilization of voltage-gated IO-inhibiting drugs has been shown to increase survival in cancer patients. Additionally, they also have the potential to be used as a therapeutic option in MS and HNDDs.	[50,115,116]
Amiloride (AM) and its derivatives: liposomal amiloride, benzamil, and bepedril	AM is a non-specific and weak NHE inhibitor and cell acidifier that has been recommended in both MS and cancer treatment. In MS, as well as in other HNDDs, AM is also recommended as a preventive measure, AM acts as an NHE, Ca^++^/Na^+^ exchanger, and ASIC inhibitor. Paradoxically, since AM is a cell acidifier, it has been shown to have protective effects in different neurodegenerative situations by preventing acidosis-induced cellular Ca^++^ injury, also preserving myelin in hypoxic and inflammatory conditions. Bepridil, a more powerful NHE inhibitor than AM, also acting as an ASIC1 inhibitor, has been reported to protect myelinated axons from degeneration.	[6,8,11,30,59,109,110,111,117,118,119,120,121,122,123,124,125,126,127,128,129,130,131,132,133,134]
Cariporide (CP)	CP, a more potent and specific NHE inhibitor than AM, protects neurons from apoptosis, attenuates glutamate-mediated mitochondrial death pathways, as well as decreases the cellular entry of Ca^++^ and the mitochondrial overloading of ROS. Thus, NHE1 inhibition may prevent neural necrosis and apoptosis. CP has also been advised to be considered in cancer treatment.	[117,135]
Other anti-MS drugs: aminopyridine (4-AP), nafamostat mesylate (NM), and butyrate	4-AP works as a potassium K^+^ channel blocker and is clinically approved to treat walking deficiencies in patients with MS. It helps to improve nerve conduction induced by demyelination. NM has been tried in cancer patients but not in patients with MS or other HNDDs.	[136,137,138,139,140,141]
Butyrate	In MS, butyrate protects the intestinal barrier, increases the Treg cell population, reduces proinflammatory T lymphocyte levels and facilitates the differentiation of oligodendrocytes, in addition to suppressing demielyination and enhancing remyelination.	[139,140,141]
PTIs and human growth factors (hGF)	Different growth factors (GFs) have been tried for neuronal protection in different HNDDs. Platelet-derived growth factors (PDGF) have been most successful in AD and PD models using rodents. They are bound to be an important part of the clinical armamentarium in MS and other HNDDs.	[26,142,143,144,145,146]
Human growth hormone (hGH)	GHs play a very important role in the development and maintenance of brain functions. The brain has been considered to be a GH-target tissue. Moreover, production of GH and its receptor occurs in neural stem cells, where the hormone induces their proliferation and differentiation. In rats, GH administration induces neural cell proliferation and recovers motor function after frontal cortex lesions. GH induces the expression of a number of neurotrophic factors. In humans, hGH administration improves cognition, learning, and memory in different pathologies. GH administration in rat AD models improves spatial cognition. The possible use of hGH in AD in humans has been postulated. In mice models of ALS, GH has a protective effect on motor neurons, increasing survival times and improving motor performance. GH concentrations are low in the CSF of ALS patients; however, its administration has no effect on the clinical progression of this fatal HNDD.	[146,147,148,149,150,151,152,153,154,155,156,157,158,159,160,161,162]
Melatonin (MT)	MT can prevent NO stress-induced mitochondrial dysfunction in experimental models of AD. In mouse models of AD, there is a significant clinical improvement after chronic MT treatment, with an improvement of cognition and memory, as well as a reduction in the deposits of Aβ. MT administration at pharmacological dosages should be considered in the adjuvant treatment of certain HNDDs, such as MS. MT seems to improve intestinal and adipose lipid metabolism in experimental MS, and seems to improve the progression of the disease.	[153,163,164,165,166,167,168]
Nitric oxide (NO), inhibitors	Nitric oxide synthase (iNOS) is increased in MS patients, supporting MS pathogenesis, mainly inhibiting the mitochondrial respiratory chain. Agmantine is an NO inhibitor that shows activity against MS in vivo, and it should be considered within the integral treatment of MS. Moreover, hydralazine decreases the accumulation of LA and is a promising drug in the complementary treatment of MS.	[169,170,171,172,173,174,175,176,177,178,179,180]
Mitochondrial booster agents in MS: methylene blue (MB), alpha lipoic acid (α-LA), and fermented wheat germ extract (FWGE)	MB restores mitochondrial function and has a role in the treatment of MS. However, despite it also being an NO inhibitor, it has not been reported to show activity against MS. FWGE is a potent mitobooster agent, restoring mitochondrial activity, and it also suppresses the Warburg effect and decreases the lactic acid (LA) burden in MS. So far, however, its utilization has been reported mainly in the cancer context.	[181,182,183,184,185]
Calcium (Ca^++^) entry inhibitors in MS	An excess of i.c. Ca^++^ increases ROS, interferes with neurotransmission and neuroinflammation, promotes further i.c. acidification, favors apoptosis, and leads to the development of MS. Administration of Ca^++^ inhibitors represents a fundamental neuroprotective measure in MS. Several of these inhibitors are clinically available. Bepridil also works as a Ca^++^ channel blocker and has been shown to induce an outstanding improvement of symptoms in the management of MS in model systems.	[16,22,186,187,188,189,190,191,192,193,194,195]
Caspase-3 inhibitors in MS	Caspase inhibition is therapeutically indicated in AD, MS, and other HNDDs. Since caspase-3 promotes pyroptosis (apoptosis associated with a high inflammatory component), suppressing pyroptosis becomes a promising strategy in the management of MS.	[30,31,196,197,198]
Glutamate lowering agents	In MS, excess glutamate levels cause the degradation of enzymes, transporters, receptors, and signaling. Thus, a therapeutic strategy being considered as a novel therapeutic approach is to minimize excess glutamate in the CNS with the glutamate oxaloacetate transaminase (GOT). Interestingly, MS is also mediated by autoimmune T cells that can produce and release glutamate.	[199]
Antilactacemics in MS	Antilactacemics offer a new therapy to minimize myelin degeneration in MS. Among them, alkaline preparations are important in the treatment of MS, since they have been shown to relieve MS patients from ocular symptoms, tiredness, and muscle pain. They have been used in the cancer context too. In combination with DMSO, sodium bicarbonate has proved to be a safe and effective treatment of pain in malignancy. Moreover, the use of oral DMSO has also been used in treating autoimmune and ocular diseases.	[15,200]

### 1.6. Ion Channel Activity in Neurodegeneration and Cancer

#### 1.6.1. Ion Channel Activity in Neurodegeneration

Proton-gated, acid-sensing ion channels (ASICs), mainly the isoform ASIC1, favor the excessive intracellular accumulation of Na^+^, Ca^++^, and H^+^ in MS and other HNDDs, resulting in severe neural damage, secondary to Ca^++^ overload and acidification-mediated apoptosis [45,46] (Figure 2). Other channel blockers inhibit T cell activity, once again placing metabolic changes before immune or autoimmune changes [201]. These ASIC1-mediated metabolic abnormalities induce axonal degeneration in the CNS of mice, further showing that tissue acidosis, which also activates ASIC1, precedes the onset of neuroinflammation and other autoimmune phenomena [58,122].

In the same vein, i.c. H^+^ concentration determines neuronal excitability through ion channel activity [110]. The higher the H^+^ concentration, the lower the pHi and the excitability. A decrease in the CNS pHi opens ASIC1, which, through the stimulation of Ca^++^ into neural cells, facilitates axonal injury, the induction of apoptosis, and, most importantly, demyelination in MS and β-amyloid accumulation in AD [110,122] (Figure 2). Spontaneous neurotransmitter release and neuromuscular transmission are reduced by ASIC1 activation at motor nerve terminals, effects that are reproduced by acid solutions [22]. Furthermore, ASIC1 activity induces autoimmune inflammation in the CNS, as well as axonal degeneration, once again placing metabolism before immunity [58]. In both the CNS and the peripheral neural system, the activation of Ca^++^-permeable channels during acidosis exacerbates neurodegeneration in mice [20]. ASIC1 activity has been recognized in many pathologies of the CNS, such as MS, Huntington’s disease (HD), and Parkinson’s disease (PD), making them a target for therapy [202]. On the contrary, neural cells lacking ASIC are resistant to acid injury [10]. Finally, the NaV1.5 Na^+^ channel is also involved in regulating endosomal acidification [203] (Table 3).

#### 1.6.2. Ion Channel Activity in Cancer

Different ion channels (IOs) are involved in the deregulation of pHi/pHe in cancer cells and, in this way, in stimulating cell proliferation and adhesion, motility, extracellular matrix invasion, resistance to apoptosis, and metastatic potential [10,11,47,48,49,50,51,52]. Consequently, IOs have been proposed as a potential target for anticancer therapy and as a new hallmark for cancer [47,48,49,50,51,52,204,205,206,207]. On the contrary, cellular acidification, which is the ultimate driver of the clinical picture of certain HNDDs, has been shown to be highly deleterious in neurodegeneration also through pH-deregulating IOs [6,8,10,11] (Figure 2 and Table 2).

There is also a group of voltage-gated Na^+^ and H^+^ channels (Hv) whose activation results in the extrusion of excess intracellular H^+^ and, therefore, participates in the acidification of the extracellular compartment [208]. Hv1 and/or Nav1.5 were found to be highly expressed in highly invasive BC cells, but not in poorly invasive BC cells [53]. Thus, their therapeutic downregulation reduces BC cell migration, invasion, tumor size, and clinical stage, all due to the induction of i.c. acidification [209,210,211], Hv1 also being an important factor favoring the CPR of cancer cells (Figure 1).

### 1.7. Human Growth Factor (GFs) Abnormalities in Neurodegeneration and Cancer

A deficiency of certain human growth and trophic factors (GFs) is directly involved in the pathogenesis of several HNDDs. Different animal and human studies have linked cognitive deficits with changes in brain and peripheral trophic factors [212]. The removal of essential GFs results in apoptosis [60]. The deficiency of different GFs is a general characteristic of HNDDs that contributes towards certain symptoms, such as the lack of tropism and mobility, of these HNDDs [61]. Since NHE1 activity is fundamental to the pH regulation of the CNS [59], different GFs, platelet-derived (PDGF) or otherwise, also normalize neural homeostasis by stimulating cellular metabolism and DNA synthesis [62,63,64] (Table 3). Therapy-wise, a wide array of GFs, platelet-derived (PDGF) or otherwise, activate NHE [62,63]. The use of plasma rich in growth factors (PDGF) has been shown to induce an important decrease in brain amyloid-β (Aβ) deposition and tau phosphorylation in a mice model of AD, while also decreasing astrocyte reactivity and synaptic loss and reducing inflammatory responses, thus promoting Aβ degradation [26].

In both hypotrophic and normal situations, GFs increase the pHi, and stimulate cellular metabolism and DNA synthesis; however, they may also have a direct carcinogenic effect [64,117,213]. Direct therapeutic possibilities arise from these pathogenetic associations, suggesting that the use of GFs can improve the therapeutic armamentarium of certain HNDDs [62,214,215]. Some authors have studied the activity or deficiency of different GFs in HNDDs, as well as their relationships to i.c. and e.c. acid–base homeostasis, all within the wide-ranging concept of the “trophic factor withdrawal syndrome” (TFWS) [6,7] (Figure 2).

Initially departing from the seminal homeostatic concept, these data further contribute towards opening new and promising areas of regenerative treatment in HNDDs. It can be concluded that the therapeutic failure in the prevention and treatment of MS and other HNDDs can be largely secondary to the scarcity of information regarding the specific roles of the different GFs in the pathogenesis of the TFWS [6,7]. Thus, all therapeutic efforts should be directed to maintain neural i.c. and e.c. acid–base homeostasis within a very narrow physiological range. This would prevent the metabolic collapse induced by neural cell acidification and Ca^++^ entry, as well as the secondary activation of cell death programs, in MS and/or other HNDDs.

### 1.8. Mitochondriopathy in MS and Other Human Neurodegenerative Diseases (HNDDs)

The highlighted interplay of mitochondrial dysfunction, hypoxia, acidification, and inflammation is a relevant issue in MS and other HNDDs [4,23,216,217].

Mitochondrial dysfunction has been considered to represent a significant factor in the etiopathogenesis and progression of ALS, AD, PD, and HD. Several lines of evidence suggest that mitochondrial dysfunction may be crucially involved in the pathogenesis of ALS [66]. Existing evidence indicates that mitochondrial disease, either as a primary cause or as a contributing factor, should become an important target for therapeutic intervention in different HNDDs [218,219]. Overall, and despite the accumulated evidence available, it can be said that the fundamental role of acidification in the onset and progression of HNNDs has been highly underestimated [220]. 

Different mechanisms are involved in impaired mitochondrial function. These include free radicals, mitochondrial inducible NO synthase activity and NO production, pH changes, a disrupted electron transport system, oxidative stress, and abnormal mitochondrial permeability [67]. Therefore, mitochondriopathy should be firmly considered in the pathogenesis and treatment of MS [67]. It is known that myelinogenesis is highly damaged during different stages of MS. This process, whether it takes place in the peripheral nervous system (Schwann cells or neurolemocytes) or the central nervous system (oligodendrocytes), is tightly associated with mitochondrial dynamics [68,69]. Therefore, any perturbation in mitochondrial function is likely to damage myelinogenesis and worsen the evolution of MS. 

Neuroinflammation is another crucial characteristic that defines MS pathology. Different cytokines are involved this process, such as IL-4, IL-10, IL-17, and TNF-α [221]. Furthermore, the stimulation of microglia and macrophages with the release of free radicals (e.g., reactive nitrogen species (RNS) and/or reactive oxygen species (ROS)), leads to mitochondrial damage by upregulating cellular death pathways. In addition, the mitochondria play a crucial role in oligodendrocyte differentiation [70]. Thus, restoring and/or stimulating mitochondrial function may result in remyelination and improve the evolution of MS.

### 1.9. The Role of Lactate in MS 

It had been shown that serum lactate (LA) measurements are higher in MS patients as compared to healthy individuals. LA levels also become elevated with disease activity, progression, and/or during relapses [71] (Figure 2)**.** Cerebrospinal fluid (CSF) LA concentrations show a close link between MS plaque activity and LA metabolism [72]. Moreover, bacterial meningitis induces neuroinflammation and neuronal damage through the release of reactive oxidative species and nitric oxide. This favors the onset of HNDDs, such as dementia [222,223,224], which can also be accompanied by elevations of LA [225,226]. Despite the unknown origin of these metabolic abnormalities, LA has been considered a biomarker of mitochondrial deregulation and a worsening agent in neuroinflammation [227,228,229,230,231,232,233,234]. However, an apparent contradiction arises from the fact that the increased secretion of LA during exercise is associated with improving vasculature [67]. This would decrease hypoxia, another one of the critical factors associated with MS, through a hypoxia–inflammation vicious cycle [235]. It can be concluded that LA in MS should be carefully monitored, as it can even improve brain physiology through a “lactate-shuttle” [236], taking into account that some brain tissues utilize LA as an alternative energetic fuel. 

### 1.10. The Role of NMDA Receptors in HNDDs

Glutamate is the primary excitatory neurotransmitter of the central nervous system and has a central role in a complex communication network established between neurons, astrocytes, oligodendrocytes, and microglia. While glutamate induces multiple beneficial and essential effects, excess glutamate is catastrophic, as it induces excitotoxicity and major loss of brain function. Synaptic dysfunction may be due to perturbed synaptic calcium handling in response to over activation of glutamate receptors, namely the N-methyl-D-aspartate receptors (NMDARs). The toxicity is principally mediated by excessive Ca^2+^ entry, primarily through the NMDARs [237,238], since NMDARs have a much higher permeability for calcium ions compared to other iGluRs [239,240]. This is a fundamental mechanism in HNDDs, including Alzheimer’s disease [73,74] and multiple sclerosis [7,8,75,76]. This is important because, while neuron degeneration and death are the ultimate consequences of MS, it is widely accepted that alterations in the function of the surrounding glial cells are key features in MS progression.

### 1.11. The Role of Gut Microbiota in HNDDs and Cancer

An important and growing research area regarding the links between neurodegeneration and cancer is the role of microbiota in neural disease etiopathogenesis. Studies conducted in recent years indicate that the composition of the human gut microbiota plays a fundamental role the etiopathogenesis of many diseases, including the development of HNDDs and cancer. Epidemiological studies have demonstrated that the composition of the intestinal flora can either facilitate or hinder the possibility of developing a HNDD or cancer. As a recent study showed, subjects developing Alzheimer’s disease or Parkinson’s disease will be somewhat protected against developing a malignant tumor, and vice versa [78]. This antagonism does not affect genetic neurodegenerative disorders, such as Huntington’s chorea or ALS, although in both diseases the composition of the intestinal flora is altered. This probably indicates that the affected nervous system also leads to gut dysbiosis, in agreement with the known relationships between the gut and the brain (the gut–brain axis) [241,242]. 

In the case of cancer, as in HNDDs, an increased intestinal permeability allows the entry of host bacteria and the harmful metabolites produced by these bacteria, as well as some neurotransmitters and intestinal hormones. This cohort of abnormalities facilitates the onset and/or progression of diverse non-genetic cancers. In summary, the available data seem to indicate that gut dysbiosis is another important metabolic link between the development of HNDDs and cancer. What both kind of diseases share is the development of intestinal inflammation, with secondary damage to intestinal permeability and impaired Treg (regulatory T cell) function and immunity [78] (Table 2). Since both opposite pathologies have a similar departure point, at least in the case of non-genetic causes, the question that now arises is: what is the cause of the etiopathogenic differences between HNDDs and cancer regarding intestinal dysbiosis? A first approach to this question comes from a recent review by Hang et al. [78]. These authors describe the effects of the six most frequent taxa of intestinal bacteria on the production of cancer or HNDDs and/or the improvement of each of these pathologies. After analyzing the results of several epidemiological studies, they concluded that, while the predominance of anticancer bacteria (*Bifidobacteria*, *Lactobacillus*, and *Blautia*) is higher in the gut of HNDDs as compared to healthy individuals, the abundances of *Prevotellaceae*, *Prevotella*, and *Ruminococcaceae* are higher in cancer patients than in healthy subjects. However, although this is well documented, there are many unsolved questions regarding the last mechanism responsible for the development of HNDDs and/or cancer. These come from the fact that both diseases share similar degrees of oxidative stress and inflammation. Therefore, where are the points at which these mechanisms diverge in order to activate one and deactivate the other?

#### 1.11.1. Gut Microbiota and the Immune System

A normal communication between gut microbiota and the immune system of the organism is key to maintain immunological homeostasis [243]. The maturation of T cells in the intestine takes place in the gut lymphoid tissue. These T cells can develop into Treg, Th1, Th2, or Th17 cells by the action of antigen-presenting cells and intestinal epithelial cells, as well as by other metabolites, such as short-chain fatty acids (SCFA) [244]. Treg cells are immune cells that play a key role in many autoimmune diseases, including MS, and the normalization of its activity seems to be very important for controlling MS. 

Treg is dysregulated in gut dysbiosis, and the result is the activation of pathogenetic factors that produce proinflammatory cytokines and chemokines. In addition, Treg dysregulation also occurs in cancer. It is known that Treg can be differentially modulated by different bacterial taxa, and also by the presence of other important intestinal metabolites, such as retinoic acid and its receptor. This might explain the activation of different cytokines, among them Il-6, depending on the pathogens responsible for gut dysbiosis. However, we still cannot explain that mitochondrial dysfunction, oxidative stress, and inflammation exist not only in different HNDDs, but also in cancer, or how this relates to the differences in i.c. pH in both pathologies. (Figure 1 and Figure 2). The possibility exists that this occurs by epigenetic mechanisms, although there are still no data that can demonstrate it. In fact, epigenetic changes modulate the transcription of some genes linked to a specific T cell lineage, consequently damaging the chromatin structure and inducing different transcriptional competence of a gene [245]. 

#### 1.11.2. Neuroinflammation in MS

In MS, there is a proinflammatory gene, Il-6, responsible for the activation of the microglia and astrocytes and the transportation of B and T lymphocytes into the brain. In addition, the Il-7 gene, which also is a strong proinflammatory factor, plays a key role in the modulation of T lymphocytes [246]. Bacterial metabolites can cross the blood–brain barrier (BBB) and activate toll-like receptor 4, which aggravates neuroinflammation and neurodegeneration. This is the case for a typical inducer of MS, the lipopolysaccharide (LPS) [247]. 

In general, the nature of MS is mostly considered to be an inflammatory autoimmune disease with some unique features, namely: (A) it is a chronic neuroinflammation involving the infiltration of lymphocytes into the CNS; (B) it involves demyelination, affecting nervous signal transmission, gliosis, axonal and oligodendrocyte damage, thus inducing a progressive neurological dysfunction [248]. 

Apart from some general factors, such as a decrease in plasma vitamin D, smoking, or a sedentary lifestyle, which seem to be epidemiologically involved, there are other important factors that appear to be involved in MS development. As stated above, an important product formed in the intestine, providing there is sufficient intake of vitamin A in the diet, is retinoic acid (RA). Vitamin A is metabolized by the intestinal epithelial cells, producing RA. Subsequently, RA plays a critical role in tipping the balance from a regulatory to an inflammatory immune response. High concentrations of RA induce the differentiation of naive T cells into Treg cells, while at low concentrations RA is key for the production of proinflammatory cytokines, such as IFN-γ and IL-17A, by T helper 1 and 17 cells (Th1, Th17). These are needed for the inflammatory immune response to infection [249,250]. From these and other studies, it can be stated that an adequate production of intestinal RA is essential for normal gut homeostasis, and for the prevention of gut dysbiosis [251]. 

#### 1.11.3. pH as an Intracellular Effector Controlling Differentiation of Oligodendrocyte Precursors (OPCs)

pH acts as a fundamental factor in controlling oligodendrocyte maturation via the activation of the ERK1/2 in pathway in culture [252]. The optimal pHi for OPC differentiation is 7.15, a value at which astrocyte differentiation is strongly reduced. Conversely, acidic intracellular pH acutely interferes with the differentiation of OPC into mature oligodendrocytes, therefore decreasing the possibility of myelination or remyelination. Finally, RA increases OPC pHi, while preventing the alkalization that follows. Therefore, RA treatment completely abolishes OPC differentiation into mature oligodendrocytes, a process that seems to depend mainly on the ERK1/2 signaling pathway, and that is more pronounced when NHE1 activity is present, as observed in vascular smooth cells [253]. This pHi-dependent effect on differentiation has been observed in many other types of cells. On this basis, it is likely that reduced gut RA production, occurring as a consequence of deficient vitamin A intake or gut dysbiosis, contributes to the loss of myelin sheaths and the absence of remyelination in MS due to the lack of fully functional oligodendrocytes, together with the inflammation and oxidative stress above described. Overall, these considerations would also explain the acidic pHi in MS and other HNDDs.

#### 1.11.4. Gut Microbiota and Short-Chain Fatty Acids in MS

Finally, gut microbiota digest dietary fibers to produce short-chain fatty acids (SCFAs), mainly acetate, propionate, and butyrate [254]. These are mainly produced by anaerobic bacteria, such as *Firmicutes*. These SCFAs participate in the regulation of intestinal permeability and also in the regulation of immune responses. They cross the intestinal barrier and are able to reach the CNS, extensively modulating a wide array of physiological functions, such as mitochondrial function, neurotransmitter production, immune regulation, and gene expression. Any significant alteration in the composition of the gut microbiota leading to the decrease in the production of these SCFAs can be involved in the pathogenesis of several HNDDs, including AD, PD, MS, as well as other CNS diseases. The increased blood–brain barrier (BBB) permeability that results from the decrease in these SCFAs allows proinflammatory cytokines and reactive oxygen and nitrogen species (ROS/RNS) to enter the brain, leading to the overactivation of microglia and damage to the brain [255]. 

## 2. On Treatment. Metabolically and Biochemically-Derived Preclinical and Clinical Treatment Proposals in HNDDs. Therapeutic Options

### 2.1. Therapeutic Approaches and Options to the Proapoptotic Metabolism of MS and HNDDs

To counteract neural acid–base abnormalities, novel strategies for the prevention and treatment of HNDDs have been proposed. These measures consist of stimulating cell metabolism through the use of a cohort of GFs and different hormones. The aim is to normalize i.c. and e.c. homeostasis by: (1) increasing neural pHi and pHe in HNDDs cells through the upregulation of dynamic buffering mechanisms, and (2) prevent and/or correct an acidotic tendency through the activation of several alkalizing proton transporters (PTs), mainly the Na^+^/H^+^ exchanger isoform 1 (NHE1) and proton pumps (PPs) [6,7,8,30,109,123,124,125]. Indeed, there is no doubt regarding the essential function of the NHE1 in regulating the acid–base homeostasis of neurons. This indicates that the presence of an upregulated NHE1 is of paramount importance in the maintenance of neuronal pHi within healthy homeostatic parameters. Among the different proton transporters, NHE1 appears to be the main mechanism that prevents neural cells from entering into a pathological decrease in pHi and pHe and, therefore, towards cellular death through cytosolic acidification [8,59]. However, other membrane-bound H^+^ transporters and pumps also play an important role in this area [18,25,124]. Surprisingly, even aging alone lowers pHi in the rat hippocampus, which suggests that aging can decrease the natural resistance to maintain a normal and healthy acid–base homeostasis in the CNS. Perhaps this occurs because time itself decreases the capacity to adapt to different stresses and energetic demands (“the stress of life” by Hans Selye) [256]. This phenomenon appears to be the mirror image of what Otto Warburg once said regarding the cancer situation, namely that “only time causes cancer” [257]. 

### 2.2. Inhibition of Ion Channels in Neuroprotection and Cancer

It has already been considered that the myelin-producing oligodendrocytes are a fundamental issue in MS regarding the repairment of damaged nerves [258]. It has also been noted that a hostile acid–base microenvironment can diminish the remyelination potential of oligodendrocytes [103]. The same authors have shown that an acidic pHe decreases the migration, proliferation, and survival of oligodendrocytes. Microenvironmental acidity also appears to be a fundamental metabolic factor inducing a decrease in remyelination, while at the same time hindering axonal regeneration and normal neurological functions [6,7]. 

From a therapeutic perspective, neural cell death induced by acidosis is inhibited by ASIC1 blockers. ASIC1 contributes to the damaging of i.c. accumulation of H^+^, Na^+^, and Ca^++^, and is overexpressed in acute MS lesions [120]. ASIC1 inhibitors, such as amiloride and its potent derivative benzamil, are considered to be effective in improving the symptoms of Huntington’s disease (HD) [13] (Section 2.4.2) (Table 3). Thus, the pharmacological targeting of ion channels [50,115,116], such as the Na_V_ channels and the acid-sensing Ca^++^-permeable channels, represent a rational strategy that can be exploited as a preventive measure in neuroprotection [20], as well as in the treatment of different HNDDs [50,115]. Importantly, there are some ASCI inhibitors clinically available nowadays that can already be used in humans. These are considered in the next Section 2.3. Finally, thorough lists of potential neuroprotective agents in MS have been published over the years [259]. In any case, it should be taken into account that any positive therapeutic findings in animal studies do not mean that the same conclusions can be directly applied to humans, as previously found for animal studies on MS, in which TNF-alpha inhibitors suggested promising results that were not further confirmed in humans. 

Bone marrow mesenchymal stem cells (BMSCs) are able to differentiate into many types of tissues, having the potential of replacing damaged neurons, promoting the reconstruction of nerve conduction pathways, upregulating angiogenesis, and decreasing apoptosis [260]. In addition, an increasing number of studies are investigating stem cell therapies in humans with MS, ALS, and other HNDDs, as well as in cancer and many other diseases. However, it is still too soon to reach definite conclusions [261,262,263]. 

### 2.3. Clinically Available ASIC Inhibitors and Other Measures in the Metabolic-Derived Treatment of HNDDs and MS

Axon remyelination in the central nervous system requires oligodendrocytes that produce myelin. The failure of this repair process is characteristic of neurodegeneration in demyelinating diseases, such as multiple sclerosis, and it remains unclear how the lesion microenvironment contributes to the decreased remyelination potential of the oligodendrocytes. Here, we show that acidic extracellular pH, which is characteristic of demyelinating lesions, decreases the migration, proliferation, and survival of oligodendrocyte precursor cells (OPCs) and reduces their differentiation into oligodendrocytes. Furthermore, OPCs exhibit directional migration along pH gradients toward an acidic pH. These in vitro findings support a possible in vivo scenario whereby pH gradients attract OPCs toward acidic lesions, although resulting in a reduction in OPC survival and motility and hindering progress toward demyelinated axons, and is further compounded by decreased differentiation into myelin-producing oligodendrocytes. As these processes are integral to OPC responses to nerve demyelination, our results suggest that lesion acidity could contribute to decreased remyelination.

The main therapeutic aim is to find further and even alternative approaches that can improve the therapeutic results in the treatment of HNDDs, and more specially of MS. This is because MS, as a neurodegenerative process, represents a unique case among HNDDs. Thus, it deserves a special study, not least due to its paradoxical sawtooth evolution, its metabolic and lactate-specific abnormalities, and its selective immune implications. To a certain extent, these special features separate MS from other HNDDs. No matter what, in any of these neurological degenerations, certain drugs are aimed at rescuing the acid toxicity induced by ASIC1, either by downregulating or by blocking it. In addition, it is most important to realize that, among the ASCI inhibitors that are clinically available nowadays, amiloride and its more potent and specific derivative benzamil, have been shown to be effective at least in improving some of the molecular characteristics of HNDDs, such as HD [13]. Along the same line, benzamil and dichlorobenzamil, as well as bepridil, also exhibit neuroprotective properties in an optic nerve model. Furthermore, bepridil has been reported to protect myelinated axons from degeneration [126] (see Section 2.4.2 and Section 2.4.3 and Table 3). 

### 2.4. Amiloride (AM) and Amiloride Derivatives in the Neurology and Oncology Clinics. Benzamil, Bepedril, Cariporide, and Other Proton Transport Inhibitors

#### 2.4.1. Amiloride (AM) Has Been Shown to Have Protective Effects in Different Neurodegenerative Situations

Amiloride preserves myelin even under hypoxic and/or inflammatory conditions [118]. This suggests that NHE1 inhibition with AM can offer a means of treating necrosis and apoptosis in neurodegeneration as a preventive measure. Blocking NHE1 activation with more specific and powerful NHE inhibitors, such as cariporide, although not yet clinically available, might provide stronger neuroprotection by protecting cells, not only from pathological increases in cellular Ca^++^ entry, but also by downregulating the production and decreasing the cellular overload of reactive oxygen species (ROS) [127]. Furthermore, inhibiting ASIC1 also decreases axonal injury and demyelization [122]. Finally, selectively blocking the Na^+^/Ca^++^ exchanger (NCX) also results in the neuroprotection of white matter [126]. 

Furthermore, the neuroprotective effects of amiloride (AM) as a non-specific NHE inhibitor as well as a Na^+^, Ca^++^, and K^+^-gated inhibitor, have been tested in patients with primary progressive MS [264]. Although this study included a small cohort of patients and extremely low doses of AM (10 mg/day), the authors concluded that AM appears to be useful in neuroprotection in patients with progressive MS. On the contrary, in other large cohort studies, AM users with MS were not associated with a decreased incidence of the disease or death [265]. It could be that the use of AM as a channel blocker has been disappointing due to its short duration of action or the low dosages utilized in these clinical studies.

Mainly in cancer research and treatment, there is a great deal of basic and animal research studies, as well as some clinical experience, on the long-term utilization of AM and its more potent derivatives [117,128,129,130]. In our research, AM has been used long-term at a dose of 10 mg, three times/daily, with occasional higher dosages up to 60 mg/day in divided doses, taken continuously over months and even years [128,130]. At dosages of 30 mg/day, AM is well tolerated. Occasionally, some degree of hyperkaliemia ensues (K^+^ up to 6 mol/L) and/or increases in BUN (up to 90 mg/dL). In these cases, AM is discontinued for two weeks and restarted at a lower dose [129,130,131]. There are other, more powerful and specific NHE and ASIC1 inhibitors that have to be taken into account; however, some of the most promising have not yet reached the clinical stage of research [10]. 

#### 2.4.2. Benzamil

Benzamil is an AM analogue with a longer duration of action than AM. Via nasal spray, it has been used for the treatment of patients with cystic fibrosis as a Na^+^ channel blocker [133]. Benzamil has also been used as a voltage-gated K^+^ channel blocker for the treatment of psoriasis, and used as topical cutaneous treatment [134]. However, despite the fact that benzamil is on the market, to date, it has not been tried in MS or any other HNDDs. It could be utilized as a repurposed drug and as a non-label drug in MS.

#### 2.4.3. Bepedril

Bepedril is a voltage-gated Ca^++^ channel blocker that has been used to treat atrial fibrillation. While it was shown to have in vitro activity against MS, it is no longer on the market as it was considered responsible for inducing ventricular arrhythmias [266,267]. Bepridil and benzamil, through their effects as inhibitors of either the Na^+^/Ca^++^ exchanger (NCX), NHE, or both, are also effective in inhibiting the growth of brain tumor cells in vitro by increasing intracellular Ca^++^ to toxic levels, thus facilitating apoptosis [266].

#### 2.4.4. Cariporide (CP)

CP has been mainly used in cardioprotection in large cardiology trials [117]. However, while this drug is easily available in a highly purified form from different chemical and pharmacological sources around the world, it is most unfortunate that has never been tested, either in clinical or preclinical oncology or neurology. A recent study using an in vitro model of excitotoxic neuronal death reported that CP, perhaps paradoxically at first, protected neurons from ischemic injury, an effect ascribed to the prevention of CNS apoptosis. In that study, CP (100 nM) attenuated glutamate-mediated mitochondrial death pathways involving the loss of mitochondrial membrane potential, as well as Ca^++^ and ROS accumulation [135]. These results further show that NHE1 inhibition offers a potential means of preventing both necrosis and apoptosis in MS and HNDDs. 

### 2.5. Other Anti-MS Drugs: Aminopyridine and Nafamostat Mesylate

#### 2.5.1. Aminopyridine (4-AP)

This drug shows some effectiveness in the control of symptoms and the course of MS [136]. 4-AP functions as a potassium K^+^ channel blocker, and it is clinically approved to treat walking deficiencies in patients with MS. It helps to improve nerve conduction induced by demyelination [137]. It is commercialized under the trade name of Ampyra (dalfampridine) and is available as 10 mg tablets. However, 4-AP is not free of potentially serious side effects. 

#### 2.5.2. Nafamostat Mesylate (NM)

NM is a serine protease and ASCI1 inhibitor that is clinically available. It is used as an anticoagulant and also to treat pancreatitis and osteoarthritis [133]. In combination with other therapies, NM has also been tried in phase I and II cancer patients [138]. As far as we are aware, it has not yet been utilized in the prevention or treatment of MS or other HNDDs.

### 2.6. Human Growth Factors (hGFs) and Platelet-Derived Growth Factors (PDGF) in MS and HNDDs

A wide array of human growth factors (hGFs) have been tried for neuronal protection in HNDDs, either induced by human growth hormone (hGH) [142,143] or derived from platelet concentrates (PDGF) (see Figure 1 in ref. [144]. Along this line, intranasal delivery of PDGF has been shown to stimulate neurogenesis and decrease neurodegeneration in mice models of AD, and also to improve cognitive function [144]. These authors have also shown that PDGF preparations induce neuroprotection in rodent models of PD [145]. However, although some relationships between PDGF, NO production, and acid–base changes in HNDDs have been described, their interrelationships are not fully understood [6,30,31]. To date, it is not known if these experimental results mediated by PDGF and/or hGH are secondary to their effect in maintaining cellular acid–base homeostasis within a physiological range or to a different mechanism [6,8,123].

### 2.7. Human Growth Hormone (hGH) in MS and HNDDs and Its Implications in Treatment

Ageing is related to the progressive decrease in the production of several neurotrophic factors, hGH among them [268]. In rats, GH administration induces neural cell proliferation [147,269] and promotes motor function following frontal cortex lesions. Importantly, hGH induces the expression of a number of neurotrophic factors (IGF-I, EGF and its receptor, EPO, VEGF, NGF), also increasing the turnover of NA (noradrenaline) and DA (dopamine) in the brain [150,151,152,153,154,268]. In mice models of ALS, GH exhibits a protective effect on motor neurons, increasing survival times and improving motor performance [155]. In addition, the peripheral administration of GH in models of AD in rats improves spatial cognition, as well as learning and memory [156]. These results suggest the possible usefulness of hGH in AD in humans, as well as in combination with other measures [157]. While hGH concentrations are low in the CSF of patients with ALS, when used alone, its administration has been described to have a positive effect on the clinical progression of this fatal HNDD [158]. 

GHs play a very important role in the development and maintenance of brain function [154]. In fact, the brain has been considered to be a GH-target tissue [270]. Moreover, the production of GH and its receptor occurs in neural stem cells, where the hormone induces their proliferation and differentiation [269]. This cerebral GH may cooperate with pituitary GH or to exogenously administered GH to increase its effect on neurogenesis. These effects have been observed in rats [269].

In humans, hGH administration has been reported to improve cognition and other neuronal alterations induced by cerebral palsy, traumatic brain injury, or old age; for instance, improved learning and memory [146,153,159,162]. 

Moreover, GH administration was able to recover a mild cognitive deficit in an elderly patient with the ApoE 4/3 genotype [271], in agreement with the results above described in a model of AD in rats [148]. 

Furthermore, GH has been shown to recover the sciatic nerve after its transection in rats, inducing the formation of a high number of myelinating Schwann cells [148]. Further still, GH treatment has been able to fully promote distal innervation in a case of caudal regression syndrome [160]. This also indicates that GH is able to induce myelination or remyelination, therefore suggesting its usefulness in the treatment of MS. Moreover, GH induces IGF-I expression, and this peptide has been shown to the promote proliferation of OPC and the differentiation and survival of oligodendrocytes [161,272]. This effect is also induced by EGF [273], the expression of which, as well as that of its receptor, may also be induced by GH.

Altogether, this data suggests that GH administration could be useful in the treatment of MS and, in fact, both the published data [274] and the preliminary data from our group show a therapeutic response in some patients, but only when the disease is not active, most likely because the role of GH in remyelination cannot occur when the oligodendrocyte microenvironment is acidic. On the other hand, the deficient secretion of GH does not seem to play any role in the development of the disease, although lower plasma GH levels have been found in patients with more severe stages of the disease [274].

### 2.8. Melatonin (MT) in MS, HNDDs, and Cancer Therapeutics

#### 2.8.1. Melatonin (MT) in MS and HNDDs

Cell death and survival are the two most critical events in neurodegeneration [6], with mitochondria being increasingly seen as an important determinant of both processes [219,275,276]. MT can prevent the NO stress-induced mitochondrial dysfunction in experimental models of AD, PD, and HD [163]. MT is a scavenger of hydroxyl, carbonate, alkoxyl, peroxyl, and aryl cation radicals, while it stimulates antioxidative enzymes, such as glutathione peroxidase and superoxide dismutase (SOD), additional to suppressing NO synthase [277]. Moreover, MT inactivates the ROS-dependent Akt signaling pathway and downregulates signals involved in pro-oxidant pathways. MT production is decreased in neurodegenerative diseases. In the case of MS, some studies have shown that the plasma and urine levels of patients taking MT are lower than in healthy age-matched controls, and that the circadian rhythm of pineal MT secretion is disrupted and more affected as the severity of the disease increases; therefore, the extrapineal mitochondrial production of MT also appears to be affected [278].

As a consequence of the oxidative stress initially produced by gut dysbiosis, and given the antioxidant properties of MT and its properties as an inhibitor of neuroinflammation, a mitochondrial protector, and its anti-apoptotic and anti-autophagic effects, treatment with this hormone has to be given serious consideration in the treatment of HNDDs [279]. The anti-inflammatory effects of MT occur by decreasing the plasma levels of proinflammatory cytokines, while its anti-apoptotic effects depend on upregulating Bcl-2 expression and decreasing caspase-3 and Bax levels [280].

Many studies have considered the potential role of MT in HNDDs, such as MS, AD, PD, and ALS. In mouse models of AD, a significant clinical improvement with chronic MT treatment at daily dosages of 10 mg/kg/day was found [281]. Furthermore, an improvement of cognition and memory, as well as a reduction in the deposits of Aβ, has been reported [164]. Importantly, MT also appears to be most beneficial treatment in other neurovascular diseases, such as Horton’s disease (“cluster headaches”), when used in high doses ranging from 50 to 350 mg/day [282,283].

In an animal model of sporadic AD in rats, MT decreases the levels of amyloid-β1-42, amyloid-β1-40, and amyloid-β1-42 [165]. These properties also make MT an important therapeutic treatment for protecting the entire organism against oxidative stress [163,166]. In summary, MT administration at pharmacological dosages should be considered in the adjuvant treatment of certain HNDDs, such as MS [168,279]. In 1992, Sandyk described the possibility of a relationship between the pineal gland and the evolution of MS [284]. In 2015, a study described the case of an MS patient that was diagnosed at the age of 28 years old. The disease and demyelination continued during the following nine years until reaching a high disability, restricting the patient to her bed or a wheelchair. At this time, she began to take MT at doses progressively increasing from 50 to 300 mg/day over four years, MT being the unique treatment. The result was a significant decrease in her disability, a fact that could only be attributed to MT [168]. We use 50–400 mg/day of MT, orally, depending on the age and the clinical situation of every patient, without measuring plasma or urine levels of the hormone, but controlling them by clinical evaluation and MRIs performed when they are needed. It is concluded that the use of MT should be seriously considered in the adjuvant treatment of certain HNDDs, such as MS. 

#### 2.8.2. Melatonin in Cancer

In the cancer context, many studies have shown the important actions of MT in the treatment of malignancy, either on its own or as an adjuvant of chemoradiotherapy [285,286,287,288,289].

In addition to its known anticancer effects as an antioxidant, MT may act on tumors epigenetically, thus increasing its therapeutic possibilities [290].

Finally, the therapeutic spectrum of MT in cancer is broadened by its protective actions against the development of mucositis or dermatitis during chemotherapy and/or radiotherapy treatments [291,292,293].

### 2.9. Treating Microbiota in Neurodegeneration

Gut microbiota has been associated with a cause–effect relationship with diseases of the CNS, mainly MS. The analysis of microbiota in HNNDs reveals a decrease in short-chain fatty acids (SCFAs). Butyrate is a short-chain fatty acid synthesized by gut microbiota. It shows promising activities in many other metabolic disorders; for example, insulin resistance, hypercholesterolemia, and many genetic metabolic diseases [294]. Treatment with butyrate in tissue culture preparations increases oligodendrocyte differentiation and remyelination [141]. Furthermore, α-linolenic acid (ALA) and valproic acid (VPA) conjugates counteract neurodegeneration and demyelination, also inducing oligodendrocyte precursor cell differentiation in MS [295]. Importantly, microbial colonization of the intestine begins shortly after birth, and it has become clear that an abnormal composition of the microbiota indicates a clear risk of developing HNDDs, and also some types of cancer. Therefore, the goal should be to achieve a normal composition of intestinal flora. Probably everyone, starting at a certain age, should perform a stool analysis to assess the composition of his/her microbiota and the relative abundance of harmful bacteria within it, in order to take the appropriate measures for its correction. Apart from this, it seems clear that eating an adequate diet, avoiding alcohol consumption and smoking, consuming vegetable fiber, having an adequate supply of vitamins (A and D, above all), avoiding stress, and carrying out regular physical activity will help our intestinal flora to achieve the optimal composition. 

In gut dysbiosis, some additional measures can be taken. Although antibiotics usually alter the normal composition of the gut microbiota, in an experimental model of MS has shown that the oral (but not intraperitoneal) administration of a specific cocktail of antibiotics (vancomycin, ampicillin, neomycin sulfate, and metronidazole) for seven days, significantly reduces the severity of experimental autoimmune encephalomyelitis (EAE), a well-established model of MS in mice, which is induced by proteolipid protein or myelin oligodendrocyte glycoprotein challenge [296]. The mechanisms by which this cocktail acts and protects from EAE suggests an induction of protective Treg cells and a reduction in the intestinal bacterial load. These led to decreased inflammatory responses. More recently, it has been demonstrated that farnesol, an organic isoprenol produced in plants and mammals, that has potent anti-oxidant, anti-inflammatory, and neuroprotective activities, and can protect against central and spinal demyelination by changing the gut microbiota composition, specifically decreasing the firmicutes/bacteroidetes ratio; however, these findings were from an animal model [297], therefore it is uncertain whether these results would be repeated in humans.

Although it has not been proved that MT can successfully treat gut dysbiosis, another recent study demonstrated that oral MT is able to reduce the production of lipopolysaccharide (LPS) by intestinal *Escherichia coli*, therefore improving intestinal and adipose lipid dysmetabolism [167]. Considering that LPS plays an important role in the development of MS, it would be interesting to study whether MT is able to regulate gut dysbiosis. 

Additionally, in recent years, it has been proposed that gut dysbiosis could be treated by means of a fecal microbiota transplant (FMT) from a healthy donor. This FMT can be carried out by means of an oral capsule containing stool or by direct colonoscopy. The advantages of FMT for treating gut dysbiosis are many, particularly if this transplant is performed occasionally. However, there is the need for a wide-scale analysis of the sample to be transplanted, since the possibility exists, as it has occurred, that this material could contain dangerous toxins proceeding from unidentified pernicious bacteria hosted in the donor. A detailed explanation of this procedure, as well as of its possible problems and future prospects, can be seen in the recent review carried out by Ser et al. [298].

### 2.10. Butyrate, Propionate, Vitamin A, and Vitamin D in MS and HNDDs

As stated before, normal intestinal production of butyrate and propionate protects the intestinal barrier, thus avoiding the entry of harmful metabolites and toxins into the host. Moreover, at the intestinal level, these SFACs regulate the immune response increasing Treg cell population. Therefore, gut dysbiosis leading to the loss of production of these SCFAs facilitates the development of HNDDs. In the case of MS, the disease is triggered by peripherally stimulated autoreactive lymphocytes that cross the BBB and transform into myelin antigens in the CNS [139]. SCFA administration, mainly propionate, increases Treg cell population levels, changes T cell differentiation to anti-inflammatory Treg cells, and reduces the level of proinflammatory T lymphocytes [140]. Moreover, it has been demonstrated that the oral administration of SCFAs recovers the demyelination induced by cuprizone and facilitates the differentiation of oligodendrocytes [141].

Consequently, it is likely that the oral administration of SCFAs may be useful for the treatment and recovery of a disease such as MS that, until now, has not achieved significant improvements with the therapies currently used. Even more, oral SCFAs can be given together with any of the current treatments for this disease. In our group, after fully analyzing the composition of the stool, we use MT and sodium butyrate (1 g/day) or sodium propionate (1 g/day). Although the disease is not fully inhibited, the interval between relapses increases and allows for remyelination by administering GH. 

Another alternative, not incompatible with what has been already described, is the oral administration of vitamin A in the diet, preferably as beta-carotenes, given its intestinal metabolization towards retinoic acid, and vitamin D (4.000 IU/day), given its immunomodulatory properties. Finally, sodium butyrate can also be useful as an adjuvant treatment in some cancers due to its antioxidant and epigenetics properties [290].

### 2.11. Nitric Oxide (NO) Inhibitors in MS

Although nitric oxide (NO) is a vasodilator, thus promoting oxygenation [169], under certain circumstances, NO and its derivative peroxynitrite (ONOO-) support MS pathogenicity through the activation of inducible nitric oxide synthase (iNOS), which is increased in MS patients [169,170]. One of the mechanisms describing how NO is associated with MS is that NO depletes the levels of cellular antioxidant agents, inhibiting the mitochondrial respiratory chain and altering other cellular metabolic pathways [171,172,173,174,175,176,177]. Agmatine is an NO inhibitor [299] that shows activity against MS in vivo when it is administered at a dose 100 mg/kg/day. Thus, this drug should be also considered in the complementary treatment of MS [178].

### 2.12. Hydralazine in MS

Hydralazine functions as a vasodilator, thus promoting oxygenation and decreasing the accumulation of LA. It has shown promising effects in the management of MS [179,180].

### 2.13. Mitochondrial Booster Agents in MS: Methylene Blue, Alpha Lipoic Acid, and Fermented Wheat Germ Extract

Some mitochondrial booster agents include methylene blue, alpha-lipoic acid, and fermented wheat germ extract [181,182,300,301].

#### 2.13.1. Methylene Blue (MB)

MB is a mitochondrial function restorative agent [182] that has a pharmacological role in the management of MS [181]. Interestingly, MB also suppresses nitric oxide (NO) production. However, some data have shown a lack of activity of MB against MS [183]. 

#### 2.13.2. Alpha-Lipoic Acid (α-LA)

α-LA is a mitobooster agent that is widely used to manage diabetic neuropathy when administered intravenously at a dose of 600 mg/day over a period of three weeks [302,303].

#### 2.13.3. Fermented Wheat Germ Extract (FWGE, Metatrol^®^)

Metatrol is a potent mitobooster agent [184] that has been widely used as an anti-aging as well as an anticancer drug in many human malignancies [184,304,305]. These authors reported that FWGE suppresses the Warburg effect, restores oxidative mitochondrial activity, and increases the carbon flux into the mitochondria. It has also been reported to inhibit in vivo tumor growth, and used in a clinical oncological context [184]. Additionally, FWGE inhibits metastatic tumor dissemination and proliferation during and after chemotherapy, surgery, and/or radiation in cancer patients, and is also beneficial in the treatment of autoimmune diseases [306]. For these reasons, FWGE has become a promising factor in the therapeutics of MS as a treatment to decrease LA burden and to control the systemic symptoms of the disease. The daily recommended dose of FWGE is two capsules/day (395 mg each) for people under 200 lb, and four capsules/day for those that weigh 200 lb or more [185]. 

### 2.14. Calcium (Ca^++^) Entry Inhibitors in MS

Calcium homeostasis is a fundamental process in cellular physiology. However, increasing i.c. calcium leads to the stimulation of a cascade of biochemical reactions that result in the formation of ROS, which stimulates apoptosis. Moreover, increasing calcium entry might be associated with increasing excitatory changes in certain neurotransmitters, e.g., glutamate, which is associated with tumor necrosis factor-alpha (TNF-α). This association can also be interlinked with transient receptor potential melastatin 2 (TRPM2), which stimulates calcium entry and neuroinflammation [186,187,188,189]. Thus, increasing calcium entry leads to the development of MS [190,191,192]. The administration of agents that interfere with calcium entry (e.g., olesoxime, quetiapine, glutathione, nimodipine, and vitamin D) represents a potential neuroprotective strategy in MS development [193,194]. Daily dosages of vitamin D in these cases range from 2000 to 5000 IU [307,308]. Finally, bepridil also works as a Ca^++^ channel blocker, and has a dramatic effect in managing MS in model systems (the dosage in mice being 3 mg/kg, subcutaneously) [195].

### 2.15. Caspase-3 Inhibitors in MS

The caspase-3 protein belongs to the cysteine–aspartic acid protease (caspase) family [309,310,311]. Caspase-3 promotes pyroptosis (apoptosis-associated with a high inflammatory component) [196,197]. Therefore, suppressing pyroptosis is a promising strategy in the management of MS [197,198]. N-acetyl-Asp-Glu-Val-Asp-7-amino-4-methyl coumarin (Ac-DEVD-amc) is an example of a caspase-3 inhibitor [312]. This compound significantly supports neuronal survival, at least in vitro [312].

### 2.16. Glutamate Lowering Agents

In MS, there are excess glutamate levels and multiple abnormalities in glutamate degrading enzymes, glutamate transporters, glutamate receptors, and glutamate signaling. Along this line, a therapeutic strategy being considered for diseases mediated by excess blood glutamate is the novel therapeutic approach to minimize excess glutamate and subsequent excitotoxicity, designed to lower excess glutamate levels in the CNS by scavenging and lowering its blood levels by intravenous injection of the blood enzyme glutamate oxaloacetate transaminase (GOT). Interestingly, MS is also mediated by autoimmune T cells that can produce and release glutamate, which then affects other cells [199]. 

### 2.17. Antilactacemics in MS

Antilactacemics have been proposed to provide a new therapeutic approach to minimize myelin degeneration in MS and other CNS disorders characterized by inflammatory demyelination [15,200]. Hydralazine also decreases the accumulation of LA. In rodents, highly positive effects in the management of MS have been shown when administered at a dosage of 1 mg/kg [179,180,313].

Most importantly, alkaline preparations appear to be a fundamental measure within the treatment of MS. At least, they have been shown to relieve MS patients from ocular symptoms, tiredness, and muscle pain [314].

A mixture of sodium bicarbonate (SB) and dimethyl sulfoxide (DMSO) has been proven to be a safe and effective treatment of pain in malignancy, probably by decreasing the extracellular acidic pH of tumors [4]. This mixture has also been clinically used as a harmless antiacid and antilactacemic measure [315,316,317,318]. The utilization of DMSO in humans has demonstrated its lack of toxicity when used for periods of up to five years. No increases in Na^+^ level, salt retention, or blood pressure changes have been observed. However, Na^+^, K^+^, and BUN should be checked at least monthly during this treatment. Recently, the use of DMSO, given orally, topically, or intravenously, has also been used to treat autoimmune and ocular diseases [319]. Therefore, it is important to know how DMSO is formulated and used:

#### Clinical Formulation and Dosages of the SB + DMSO Mixture

Thirty-four percent DMSO (99.9% pharmaceutical quality/99.9% purity), 64% double-distilled water, and 2% SB. Dosage: 10–30 mL, orally, twice a day, on an empty stomach and separated from other medications. Only crystal bottles or high-density polyethylene (HDPE). 

## 3. Conclusions

This wide-ranging approach to homeostasis, allostasis, and their deregulation, allows us to transform a general and non-specific factor, such as the pH of cells and their microenvironment, into the most specific etiopathogenic parameter in both cancer and HNDDs. This original “both sides now” perspective represents a radical change in other modern approaches to health and disease, departing from some of the more reductionistic concepts regarding the etiopathogenesis and therapeutics of different diseases and degenerative processes. Such an all-embracing perspective points towards a unified theory of the dualistic apoptosis–anti-apoptosis machineries and the pro-death and anti-death mechanisms in cellular deregulation. This paradigm hierarchically integrates the utilization of different levels of understanding, from basic to clinical research, from molecular biology to biochemistry and metabolism, and from etiopathogenesis to treatment.

As a result of this new theoretical approach, we have considered the integrated utilization of wide array of therapeutic measures in MS and HNDDs, as we have previously achieved in the field of cancer. The final aim of these efforts are: (a) improving the treatment of HNNDs, especially MS, in order to prevent, and if possible, reverse, the acidification-dependent neural toxicity and progressive apoptosis characteristic of neurodegeneration, and (b) stimulate cellular metabolism in order to recover cell and microenvironmental homeostasis and so prevent and treat the metabolic collapse and cellular death occurring in MS and HNDDs.

Interestingly, and although this may appear as a paradox at first sight, despite the fact that cancer and HNDDs present themselves at all levels of study as opposite processes when based upon their cellular pH-deviations and their respective hydrogen dynamics, some of the medications advised for HNDDs also are indicated in cancer treatment.

## Figures and Tables

**Table 1:** **pHi and pHe in normal cells, HNDDs neurons, and cancer cells during apoptosis and anti-apoptosis** [4,6,10]. Abbreviations: pHi, intracellular pH; pHe, extracellular pH; HNNDs, human neurodegenerative diseases; CPR, cancer proton reversal; TFWS, trophic factor withdrawal syndrome. For further details, see text.

Normal Cells	HNDDs Neurons	Cancer Cells
(pHi < pHe)	(Low pHi, low pHe)	(pHi > pHe)
pHi: 6.99–7.05	pHi: 6.2–6.8 (acid) (↓pHi pathological apoptosis)	pHi: 7.2–7.8 (alkaline) (↑pHi pathological anti-apoptosis) (CPR)
pHe: 7.35–7.45	pHe: 6.0–6.8 (acid) (↓pHi pathological apoptosis) (TFWS)	pHe: 6.0–6.8 (acid) (↓pHi therapeutic apoptosis)
	Acid pHi/Acid pHe	Alkaline pHi/Acid pHe
